# Dietary Supplementation with Amaranth Protein Isolate Modulates the Gut Microbiota in Children with Overweight and Obesity: A Nonrandomized Trial

**DOI:** 10.3390/nu18111690

**Published:** 2026-05-26

**Authors:** Ana P. Barba-de la Rosa, Samuel Treviño, Cesaré Ovando-Vázquez, Antonio De León-Rodríguez, Oscar de Jesús Calva-Cruz, Alberto Barrera-Pacheco, Eduardo Espitia-Rangel

**Affiliations:** 1 Molecular Biology Division, Potosino Institute of Scientific and Technological Research A.C., Camino a la Presa San Jose 2055, Lomas 4a Seccion, San Luis Potosí 78216, Mexico; aleonr@ipicyt.edu.mx (A.D.L.-R.); oscar.calva@ipicyt.edu.mx (O.d.J.C.-C.); alberto.barrera@ipicyt.edu.mx (A.B.-P.); 2 Institute of Physiology, Meritorious Autonomous University of Puebla, Prolongación De la 14 Sur 6301, Cd. Universitaria, Puebla 72592, Mexico; samuel_trevino@hotmail.com; 3 SECIHTI-National Supercomputing Center, Potosino Institute of Scientific and Technological Research A.C., Camino a la Presa San Jose 2055, Lomas 4a Seccion, San Luis Potosí 78216, Mexico; cesare.ovando@ipicyt.edu.mx; 4 Instituto Nacional de Investigaciones Forestales Agrícolas y Pecuarias, Campo Experimental Valle de México, Km 13.5 Carr. Los Reyes-Texcoco, Texcoco 56250, Mexico; espitia.eduardo1957@gmail.com

**Keywords:** amaranth protein isolate, children, insulin resistance, adiponectin/leptin, gut microbiota, overweight, obesity

## Abstract

Background: Overweight and obesity are chronic diseases that result from complex interactions including genetics, environment, eating behaviors, and limited access to a healthy diet. Amaranth protein (AmProt) has several health benefits, but no studies have examined its effects on the modulation of children’s gut microbiota. The work aimed to analyze serum levels and changes in gut microbiota in children aged 8–10 years with different body mass index (BMI) values after supplementation with AmProt. Methods: Participating children were allocated into three groups according to their BMI: normal weight (NW), overweight (OW), and with obesity (OB). Children received AmProt for 90 days. Levels of fasting blood glucose, cholesterol, triglycerides, and insulin were analyzed before and after diet supplementation. HOMA-IR and adinopectin/leptin ratio were evaluated. Feces were collected and metagenome analysis was carried out. Results: No changes in glucose levels were observed across groups and treatments; however, cholesterol and triglycerides levels tended to decrease. The HOMA-IR value increased in relation to BMI and no changes were observed after treatment. Firmicutes were highly abundant in all groups. The lower abundance of *Ruminococcus* was observed in the OW and OB groups. In the OW group, *Blautia*, *Butyricicoccus*, and *Roseburia* were also observed in increased abundance. In all groups, AmProt consumption tended to increase the abundance of *Coproccus*, *Prevotella*, and *Collinsella.* Conclusions: Supplementation of the children’s diet with AmProt showed an improvement in serum cholesterol and triglyceride levels, which could be related to changes in the microbiota related to lipid metabolism.

## 1. Introduction

Overweight and obesity are multifactorial diseases arising due to genetic, socio-demographic, and environmental factors and sedentary lifestyles. This is aggravated by the rise in consumption of high-calorie processed foods [1,2]. Obesity is linked to chronic disorders such as atherosclerosis, diabetes, and cardiovascular disease, and has a significant association with the progression of cognitive dysfunction [3]. Gut microbiota and its metabolites such as short-chain fatty acids (SCFAs) influence nutrient absorption and host metabolism, modulate gut permeability and energy harvest, and regulate the gut–brain communication; consequently, gut microbiota dysbiosis significantly contributes to childhood obesity [4,5].

Dietary interventions in combination with exercise represent a key approach to treating obesity. High-protein diets may be beneficial for the prevention and treatment of obesity [6]. Protein can maximize lean body mass retention during weight loss and stimulates the release of intestinal peptide hormones GLP-1 and YY peptide, which have anorexigenic properties, thereby reducing food consumption and energy intake [6]. However, protein quality determined by the composition of the essential amino (particularly leucine, which is a key stimulus for muscle protein synthesis) may be relevant for the prevention and treatment of obesity [6]. In addition, protein digestibility and absorption greatly influence the amount of protein that reaches the colon, where the gut microbiota can further metabolize undigested proteins/peptides, generating microbial metabolites such as SCFAs [7]. Despite this, there are few studies investigating the effect of high-quality protein supplementation on obesity status in children [6].

Amaranth seed proteins have gained renewed interest because they provide a well-balanced profile of essential amino acids and branched-chain amino acids (leucine, isoleucine, and valine). Furthermore, amaranth proteins are gluten-free and contain encrypted peptides with multiple biological functions, making them one of the most promising sustainable protein sources [8]. However, no studies have been reported on amaranth’s health-promoting effects due to the modulation of the gut microbiota. The present work aimed to conduct a prospective nonrandomized trial of the effect of amaranth protein consumption on clinical and gut microbiota changes in Mexican children aged 8–10 years with different body mass index values. Blood and stool samples were taken at the beginning of the trial and after three months of amaranth protein consumption. Results provide data that open up a new avenue for understanding the mechanisms by which amaranth proteins exert their health benefits, namely by modulating the gut microbiota.

## 2. Materials and Methods

### 2.1. Preparation and Characterization of Amaranth Protein Isolate

Amaranth flour was defatted and protein (AmProt) was extracted following the solubilization/precipitation protocol [9]. AmProt was freeze-dried and stored at 4 °C until use. AmProt was analyzed in accordance with the Official Mexican Standards (NOM-051-SCFI/SSA-2010) and characterized by SDS-PAGE, and proximate composition was determined using the standard protocols [9]. Minerals were determined using an Inductively Coupled Plasma Optical Emission Spectroscopy system (Lanbama Lab., IPICYT, San Luis Potosí, Mexico).

### 2.2. Ethical Considerations and Participant Recruitment

This study was approved by the Research Ethics Committee of the Servicios de Salud, San Luis Potosi (number of approval: SLP/006-2018, dated 1 June 2018), and was conducted in accordance with the Helsinki Declaration (2000). Informed assents were obtained from the participating children, and informed consent from their parents or legal representatives. The trial was conducted from March to July 2020. A total of 56 unrelated children, 8–10 years old (30 male and 26 female) were selected from an elementary school in Mexquitic de Carmona, S.L.P., Mexico. The height and weight of each volunteer were measured and grouped according to its Body Mass Index (BMI) [10]. A diagnosis of overweight or obesity is made by measuring people’s weight and height and calculating the body mass index (BMI) reported as kg/m^2^. In pediatric populations, weight status is not defined by fixed BMI thresholds as it is in adults. The two most widely used international standards are provide by the World Health Organization (WHO) and the Centers for Disease Control and Prevention (CDC), also known as Z-scores, to account for the rapid growth spurts that occur between ages 8 to 10, considering normal weight between −2 SD and +1 SD, overweight greater than +1 SD (equivalent to BMI = 25 kg/m^2^ at age 19), and obese greater than +2 SD (equivalent to BMI 30 kg/m^2^ at age 19). Meanwhile, the CDC uses percentiles based on growth charts defining normal weight as the 5th percentile to less than the 85th percentile, overweight as the 85th percentile to less than the 95th percentile, and obesity as the 95th percentile or greater [1,2].

Inclusion criteria included children who had not received antibiotics, prebiotics, or other drugs. Exclusion criteria included children with other diseases and those who refused to sign an informed consent form. Each group consumed 2 g of AmProt per day for 90 days. The research design is shown in Supplementary Figure S1. The final population was composed of NW (n = 8), OW (n = 6), and OB (n = 7), which was due to desertion due to the pandemic situation or to non-adherence to the treatment. During the intervention, the parents or legal representatives supervised the daily administration of AmProt. Blood and stool samples were taken before and after the study. The primary outcomes were the analysis of gut microbiota composition. The secondary outcomes were hematological and biochemical serum analyses and measurements of fecal SCFAs levels.

### 2.3. Serum Biochemical Tests

Blood samples (10 mL) were collected early in the morning under fasting conditions using BD Vacutainer^R^ without anticoagulant for hematological tests and BD Vacutainer with serum separator tube and clot activator. Blood samples were kept on ice and transported to the lab for processing (Iktan Labs, San Luis Potosi, Mexico). Serum was obtained by centrifugation at 8 °C for 5 min at 1300× *g* in a Rotanta 460R (Hettich Lab Technology, Tuttlingen, Germany); the serum was quickly separated and aliquoted into Eppendorf tubes and frozen at −80 °C until used to avoid glycolysis and glucose degradation. Hematocytometry was performed on an automatic system, Kontrolab Model 5H+ (KontroLabCo., Ltd., Giudornia, Roma, Italy).

Serum glucose, triglycerides, total cholesterol, uric acid, urea, total protein, albumin, total bilirubin, and direct bilirubin concentrations, as well as aspartate aminotransferase (AST), alanine aminotransferase (ALT), gamma glutamyl transferase (GGT), alkaline phosphatase (ALP), amylase, lipase, creatine kinase, and lactate dehydrogenase activities, were determined using commercial kits (SPINREACT, Girona, Spain) on a A15 analyzer (BioSystems, Barcelona, Spain).

Serum levels of TNF-α, IL-β, IL-6, MCP-1, IL-10, insulin, leptin, and adiponectin were quantified using commercial ELISA Kits and a Multiskan-GO microplate spectrophotometer (Thermo-Fischer Sci., Waltham, MA, USA). The adiponectin/leptin ratio was calculated, and the standard formula for calculating the homeostatic model assessment of insulin resistance (HOMA-IR) [11] was used as follows:

$$HOMA-IR=\;\frac{\left[Fasting\;Insulin\;\left(µ\frac{IU}{mL}\right)\;\times \;Fasting\;Glucose\left(\frac{mg}{dL}\right)\right]}{405}$$

### 2.4. Fecal Sample Collection

Fecal samples were obtained immediately after defecation and collected aseptically in a sterile stool container. Samples were transported to the laboratory at 4 °C, homogenized, aliquoted, and immediately stored at −70 °C until further processing.

#### 2.4.1. Measurement of Fecal Short-Chain Fatty Acids (SCFAs)

One aliquot of fecal sample (250 mg) was homogenized in one mL of H_2_SO_4_ (0.5 mmol/L) and mixed at 1400 RPM for 3 min (Thermomixer Compact 5350, Eppendorf, Ham, Germany). The homogenized sample was incubated for 20 min in an ice-water bath and centrifuged at 4 °C and 13,000× *g* for 15 min. The supernatant was recovered and subsequently centrifuged as before. The procedure was repeated twice for clarification, and then the sample was filtered through a 0.22 μm Millipore filter (Merck, Darmstadt, Germany) before injection into the chromatographic system. Analysis of SCFAs was performed using an Agilent 1100 (Hewlett Packard, Santa Clara, CA, USA) and a Rezex ROA LC Column 150 × 7.8 mm (Phenomenex Inc., Torrance, CA, USA). The mobile phase was composed of H_2_SO_4_ (0.5 mmol/L). The column temperature was 60 °C; the flow rate was 0.5 mL/min; and measurement was performed using a RID-10A RI detector. Calibration curves were performed from 0.18 to 1.8 mg/mL for acetic acid, 0.08 to 0.8 g/L for propionic acid and 0.11 to 1.1 g/L for butyric acid (6 concentration levels).

#### 2.4.2. Fecal DNA Extraction and Integrity Verification

A second aliquot of fecal matter was diluted with 1300 µL of saline solution (0.85%), and coarse debris was removed. The decanted sample (600 µL) was transferred to a new tube and resuspended. The suspension was centrifuged at 10,000× *g* for 10 min at 4 °C, and the pellet was washed twice in 1 mL of cold saline solution. The resulting pellet was used for DNA extraction according to the DNeasy UltraClean Microbial Kit (QIAGEN, Hilden, Germany) protocol. DNA was quantified using a NanoDrop One (Thermo Fischer Sci., Waltham, MA, USA), and integrity was verified by visualization on a 1% agarose gel. For microbiome analyses, the V4–V5 region of the *16S rRNA* gene was amplified using universal bacterial primers 515FB: 5′-GTGYCAGCMGCCGCGGTAA-3′ and 926R: 5′-CCGYCAATTYMTTTRAGTTT-3′. Samples were sequenced on the Illumina MiSeq platform (Illumina, San Diego, CA, USA) using 300 + 300 bp paired-end sequences. Samples were sequenced at the Integrated Microbiome Resource (IMR) (Dalhousie University, Halifax, NS, Canada).

#### 2.4.3. Amplicon Sequence Variant Inference

The R package DADA2 v1.16.0 was used to process the *16S RNA gene* sequencing data. Sequences were trimmed to 20 nt to the right side of the forward fragments and 60 nt to the right side of the reverse fragments using the *filterAndTrim()* function with default parameters. The *learnErrors()* function with default parameters was used to identify the error rates. The filtered fragments were merged using the *mergePairs()* function and chimeras were removed using the *removeBimeraDenovo()* function with default parameters. The taxonomy for each sequence was assigned using the *assignTaxonomy()* function and the silva_nr99_v138 database. From these results, we obtained the Amplicon Sequence Variant (ASV) quantification and their phylogenetic assignment.

#### 2.4.4. Diversity Quantification and Functional Prediction

Alpha diversity, as measured by the Shannon and Simpson indices, was calculated with the *diversity()* function from the vegan v2.5.6 R package. To determine the structural variation in microbial communities, beta diversity and NMDS plots were generated with custom R scripts, using Bray–Curtis dissimilarity calculated with the *ordinate()* function from the vegan package. Functional prediction of the 16S sequencing data was performed using Tax4Fun2. To predict the functional profiles of the quantified ASVs, the *runRefBlast()* and *makeFunctionalPrediction()* functions were used with default parameters and RF99NR as the reference. The pathway prediction table (pathway scores per library) was used to perform a sparse Partial Least Squares Discriminant Analysis (sPLS-DA). The *spls-da()* function from the R package MixOmics with default parameters and *ncomp = 2* was used. The plots representing the DA results were generated using custom R scripts. Correlation between selected taxa and metabolic pathways was performed with the *cor()* function; method = “spearman” from the stats R package. Variables were scaled (z-score) using the *scale()* function, before correlation calculation.

### 2.5. Statistical Analysis

Statistical analysis of results was performed on Prism 8.0 for Mac (GraphPad Software, San Diego, CA, USA). D’Agostino and Pearson and Shapiro–Wilk tests were used to assess if the dataset followed a normal distribution. For data with normal distribution, one-way ANOVA followed by Bonferroni’s post hoc test was performed. Kruskal–Wallis followed by Dunn’s post hoc test was performed if the data were not normally distributed, at a level of significance of *p* < 0.05.

## 3. Results

Amaranth protein isolate (AmProt) showed the electrophoretic profile of total amaranth proteins (Supplementary Figure S2A). AmProt contained 71% protein, 2.1% fat, and high mineral (ashes) contents (Supplementary Table S1), which were rich in Ca, Mg, Mn, Na, Si, and Zn (Supplementary Table S2). AmProt-tryptic peptides showed 60% inhibition for DPP-IV activity (Supplementary Figure S2B) and up to 70% of ECA inhibition (Supplementary Figure S2C).

### 3.1. Changes in Body Mass Index and Serum Biochemical Levels After AmProt Consumption

**Figure 1:**
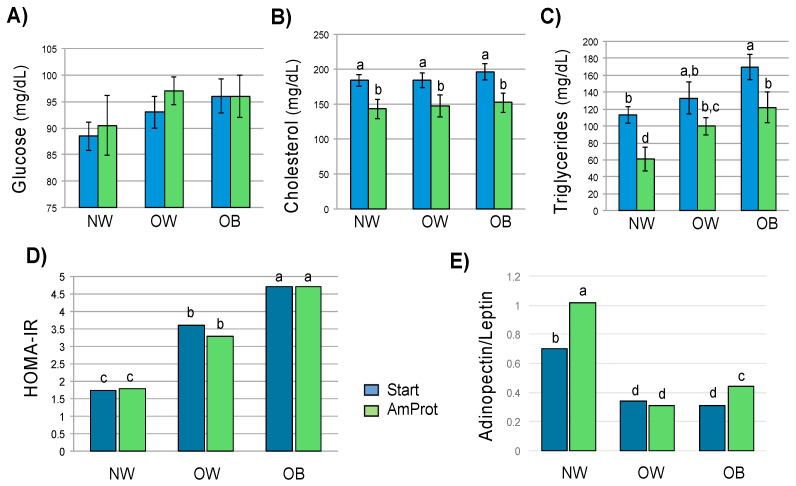
Serum biochemical levels of children with different body mass index values: (**A**) glucose, (**B**) cholesterol, and (**C**) triglyceride levels. (**D**) HOMA-IR index; (**E**) adiponectin/leptin ratio. One-way analysis of variance (ANOVA) and Holm–Sidak post hoc analysis (*p* < 0.05) was performed. The values express the mean ± standard deviation. Blue bars represent the values at the start of the trial. Green bars show the values after 3 months of AmProt consumption. The same letters above bars indicate no significant differences among mean values. NW = normal-weight group; OW = overweight group; OB = obesity group; AmProt = amaranth protein isolate.

As shown in Supplementary Table S3, children were grouped according to the BMI [10]; children with a BMI of 16.7 ± 1.3 kg/m^2^ were grouped into the normal weight group (n = 18); the overweight group (n = 12) was for a BMI of 20.5 ± 1.4 kg/m^2^; and children with a BMI of 24.5 ± 2.7 kg/m^2^ were placed in the obesity group (n = 18) as shown in Supplementary Table S3. After the 3 months of diet supplementation with AmProt, no significant changes were observed (Supplementary Table S3). Blood glucose levels in all groups ranged within normal biological limits from 88.5 to 96 mg/dL (Figure 1A; Supplementary Table S3). The same levels of cholesterol were observed in all groups (from 88.5 to 96 mg/dL) but higher levels of triglycerides were observed in the OB group (170 ± 15.1 mg/dL). Interestingly, after AmProt consumption, levels of both lipids decreased in all groups (Figure 1B,C; Supplementary Table S3).

Fasting insulin levels in children are typically 10–17 µIU/mL and elevated values are associated with physiologic or pathologic hyperinsulinemia (insulin resistance). In our study, only the OB group showed higher insulin levels than normal values (Supplementary Table S4). The calculated HOMA-IR index values were 1.7 for the NW group, 3.5 for the OW, and 4.7 for the OB group. No changes in HOMA-IR were observed after AmProt consumption (Figure 1D).

### 3.2. Muscle, Pancreas, Kidney, and Hepatic Function Tests

Biomarkers for the muscle (LDH and CK) and pancreas (amylase and lipase) showed no significant differences among groups or after AmProt consumption; all values were within the normal ranges (Supplementary Table S3). Uric acid (~5.1 to 5.5 mg/dL) and urea (19.9 to 24 mg/dL) values were within normal value ranges (Supplementary Table S3).

Regarding liver function, total protein in all groups was higher than the normal range (8.0 to 8.2 g/dL), but after AmProt consumption, these values decreased to what was normal (7.2 to 7.8 g/dL) (Supplementary Table S3). The albumin-to-globulin (A/G) ratio remained between 2.0 and 2.4 before and after AmProt consumption (Supplementary Table S3). The levels of AST, ALT, ALP, total bilirubin, and conjugated bilirubin were not different among groups, even after AmProt consumption (Supplementary Table S3). GGT values in the NW and OW groups were within normal limits (<20 mg/dL); however, the OB group showed higher values (27.2 ± 2.9 U/L) and remained elevated (28.5 ±3.1 U/L) after AmProt consumption (Supplementary Table S3).

### 3.3. Interleukin and Adipokine Levels in Serum

Lep values increased with BMI; 5.1 ± 2.7; 8.6 ± 3.9, and 11.9 ± 2.3 ng/mL for the NW, OW, and OB groups, respectively. No significant changes were observed after AmProt consumption, but although in all cases Lep levels were in the reported normal range for children, they showed a tendency to increase with BMI reaching values reported for obesity (14.5 ng/mL). On the other hand, Adpn values showed a tendency to decrease with BMI (4.8 ± 2.3 for NW, 3.0 ± 1.0 for OW, and 3.7 ± 2.5 for OB). Adpn values of 4.5 µg/mL are considered normal, whereas values of 3.5 µg/mL are related to overweight/obesity. AmProt consumption had no effect on Adpn values (Supplementary Table S4). However, the Adpn/Lep ratio changes significantly in the NW group, from 0.94 before to 1.02 after AmProt consumption. In the OW group, this ratio did not change AmProt consumption, while the OB group showed an increase after AmProt (Figure 1E).

TNF-α levels were 32 ± 19 pg/mL for NW, 23 ± 11 pg/mL for OW, and 29 ± 15 pg/mL for OB groups. After AmProt consumption, a tendency to decrease in the TNF-α in all groups was observed (Supplementary Table S4). The lowest MCP-1 values were detected in the OB group (77 ± 45 pg/mL), but in all groups, AmProt consumption resulted in a tendency to increase up to 211 ± 97 pg/mL, observed in the OW group (Supplementary Table S4). No changes were observed in either IL-1β (from 1.0 to 1.4 pg/mL) or IL-6 levels (from 0.8 to 1.4 pg/mL). However, after AmProt consumption, a tendency toward increasing IL-10 levels was observed, in NW from 5.7 ± 3.0 to 8.5 ± 4.6 pg/mL; in OW from 4.8 ± 2.3 to 9.1 ± 2.7 pg/mL; and in OB from 5.5 ± 4.4 pg/mL to 7.0 ± 4.4 pg/mL (Supplementary Table S4).

### 3.4. Short-Chain Fatty Acids

Although no significant differences in SCFAs were observed among groups, a tendency was noted. The acetic acid level was lower in the OW group but after AmProt treatment, it reached the level of the NW group (Supplementary Figure S3A). Propionic and butyric acid showed a tendency to increase in the OB group (Supplementary Figure S3B,C). In relation to total SCFAs, a significant increase was observed in the OB group (Supplementary Figure S3D).

### 3.5. Metagenome Analysis and Bacterial Community

**Figure 2:**
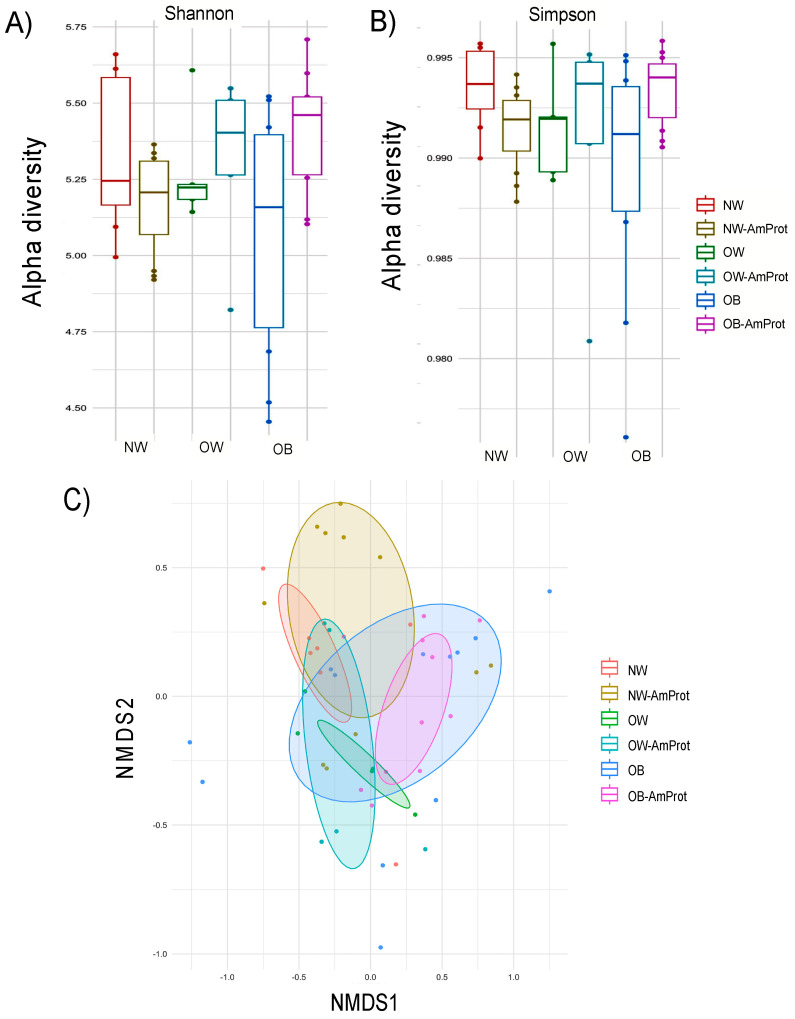
Alpha diversity represented by the (**A**) Shannon and (**B**) Simpson indices. Boxes express the interquartile range; bars express the minimum and maximum values. A Kruskal–Wallis test and Dunn’s *post hoc* analysis (*p* < 0.05) were performed. (**C**) Beta diversity as NMDS plot. NW = normal weight group; OW = overweight group; OB = obesity group. AmProt = amaranth protein isolate.

After processing the raw metagenome, data resulted in validated ASV sequences for the groups at the beginning and after 3 months of AmProt consumption. ASVs for NW were 71,725 and 68,125; for OW, they were 79,426 and 76,751; and for the OB group, ASVs were 85,999 and 85,526 (Supplementary Table S5). The rarefaction curves constructed from ASVs reached saturation, indicating reads were sufficient to represent bacterial diversity across the three tested groups (Supplementary Figure S4). Alpha diversity and Shannon indices tend to decrease with BMI, but after AmProt consumption, OW and OB groups showed a tendency to increase in the NW group (Figure 2A,B). High Shannon index values indicate high bacterial community richness or a greater number of different bacterial species. Simpson values near to one indicate that species are distributed more evenly; that means there is an equal representation of all species in the samples.

Beta diversity, as measured by NMDS plots based on Bray–Curtis dissimilarity, which measure the species turnover, showed that at the start of the trial, the NW (red oval) and OB groups (green oval) had a good separation of species or more dissimilar, but the OB group showed higher diversity (blue oval) that crossed with the NW and OB groups (Figure 2C). After AmProt consumption, beta diversity in the NW (brown oval) and OB groups (blue-green oval) tended to be more similar, but the diversity of the OB group (pink oval) tended to separate from the other samples (Figure 2C).

### 3.6. Changes in Children’s Gut Microbiota at the Phylum and Family Levels

**Figure 3:**
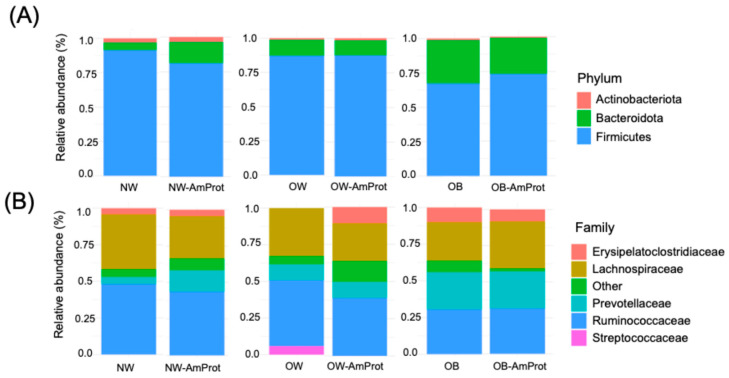
Relative abundance of bacteria at (**A**) phylum and (**B**) family levels among children with different BMI and before and after amaranth protein isolate (AmProt) consumption. NW = normal-weight group; OW = overweight group; OB = obesity group.

Firmicutes (*Bacillota*) was the most abundant phylum in all groups. In the NW group, this taxon represented ~95% of the community, but after Am-Prot consumption, Bacteroidetes (*Bacteroidota*) abundance increased. In the OB group, *Bacteroidota* abundances were higher than in the NW group, reaching up to 25%. Actinobacteria (*Actinomycetota*) species were observed, but they were less abundant (Figure 3A). At the Family level, within the *Clostridia* class, *Ruminocococcaceae* had an abundance of ~50% in the NW group, followed by *Lachnospiraceae* at ~40%. After AmProt consumption, the NW group showed an increase in *Prevotellaceae*. In the OW group, no *Erysipelatoclostridiaceae* species were observed, but this family increased after AmProt consumption (Figure 3B).

### 3.7. Significant Changes in Abundance at the Genus Level in Children’s Gut Microbiota Directed by BMI and AmProt Consumption

**Figure 4:**
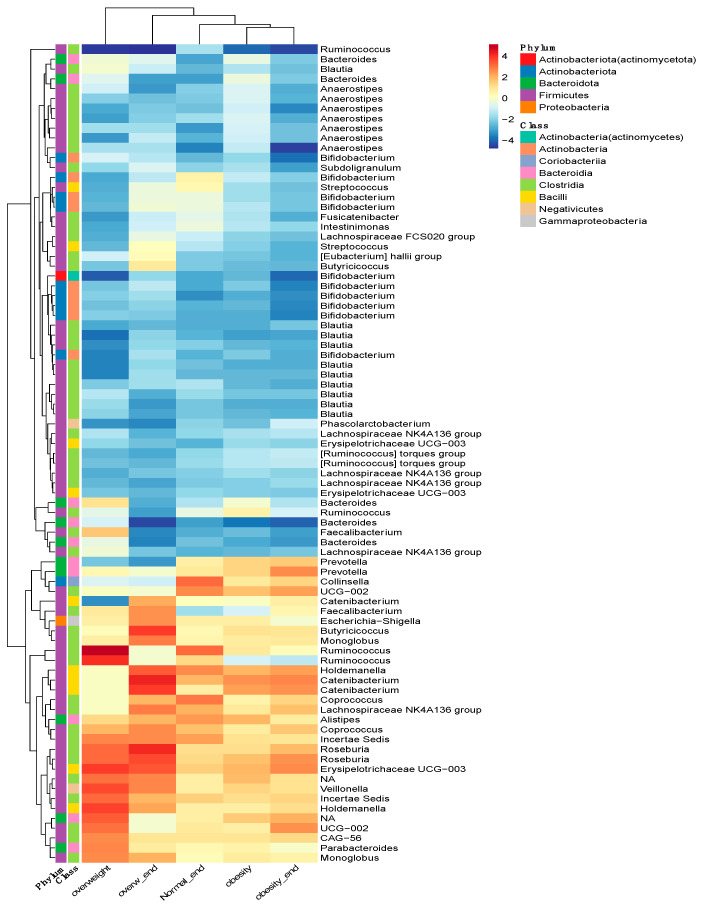
Differentially abundant genus driven by BMI and amaranth protein supplementation. Significance was defined by false discovery rate (FDR) < 0.01, *p* < 0.05, counts per million (CPM) > 5, and LogFC above 2.5.

At the genus level, several ASVs were identified as *Bifidobacterium*, *Blautia*, *Subdoligranulum*, *Bacteroides*, *Faecalibacterium*, *Roseburia*, and *Prevotella*, which were the most representative genera in the children’s gut microbiota. Significant changes, with logFC ≥ 2.5 and FDR ≤ 0.001, when comparing the groups against NW are shown in Figure 4. No changes were observed in *Bifidobacterium* and *Streptococcus* in NW-AmProt, but both were in lower abundance in the OW and OB groups, groups in which some ASVs identified as *Ruminococcus* were observed in very low abundance (Figure 4). NW-AmProt was characterized by increased abundance of *Collinsella*. In all groups, ASVs whose abundance increased after AmProt included *Prevotella* and *Holdomanella*, and a decrease in Bacteroides was observed. In the OW, NW-AmProt, and OB-AmProt groups, *Anaerostipes* decreased, while *Coprococcus* increased only in OW-AmProt and NW-AmProt. The OW was the group with the most changes observed in gut microbiota. Some ASVs for *Blautia*, *Streptococcus*, *Fusicatenibacter*, *Intestinimonas*, and *E. halii* showed a low abundance, while a significant increase in some ASVs identified as *Ruminoccoccus*, *Erysipelotrichaceae* UCG-003, *Veillonella*, and *Holdemanella* was observed (Figure 4). OW-AmProt showed increases in *Catenibacterium* and *Faecalibacterium*, *Escherichia-Shigella*, *Monoglobus*, *Butricicoccus*, and *Roseburia*.

### 3.8. Prediction of Metabolic Activities of Gut Microbiota in Children with Different BMI

**Figure 5:**
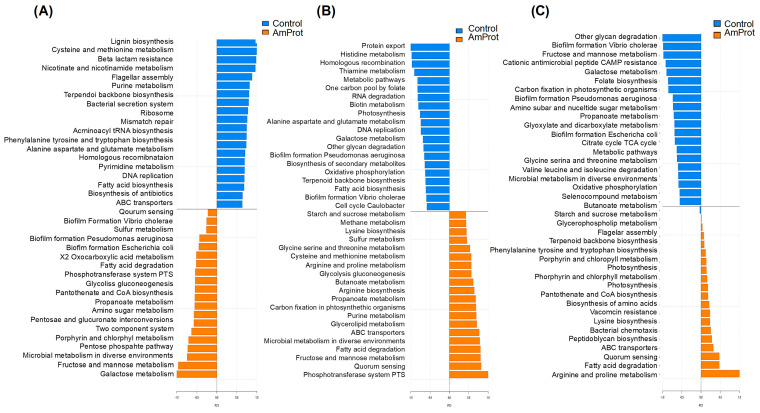
Prediction of metagenome functional pathways using Tax4Fun. Plots show changes in metabolic pathways between control group and after consumption of amaranth protein isolate (AmProt). (**A**) Normal weight; (**B**) overweight; (**C**) obesity. Blue bars showed data after, and orange bars before, AmProt consumption.

The gut microbiota profile of the NW group was characterized by biosynthesis pathways of non-essential (aspartate, glutamate, arginine-proline) and essential amino acids (lysine, cysteine, methionine, glycine, serine, threonine, phenylalanine, and tryptophan, alanine). Terpenoid and bacterial secretion system biosynthesis, fatty acid metabolism and antibiotic biosynthesis were observed to increase after AmProt consumption (Figure 5). Analysis of Spearman correlations revealed several associations between specific bacterial genera and metabolic pathways (Supplementary Table S6; Supplementary Figure S5).

## 4. Discussion

Clinical trials have demonstrated that consuming protein not only reduces body weight but also enhances body composition by decreasing fat mass while preserving fat-free mass [6,7]. AmProt showed the characteristic pattern for total amaranth proteins [9] and, upon digestion, encrypted peptides were released with high activity against DPP-IV and ECA, which exhibits several health benefits [8,9]. AmProt also contains several essential minerals with key roles in bone health, immune function, nerve function, blood pressure control, and lipid metabolism [12]. Although there are some limitations of the study, including the lack of recording of macromolecule, fiber, and calorie intake, as well as physical activity monitoring, the AmProt supplementation showed some changes in the evaluated parameters that support the beneficial effects on health reported in in vitro and animal studies.

Fasting blood glucose levels in all groups and after AmProt consumption were within the normal value ranges for children. Reductions in cholesterol and triglycerides were reported in obese mouse models when whole amaranth was administered [13]. Here we observed a decrease in both lipids after AmProt consumption. HOMA-IR index and Adpn/Lep ratio showed that only the OB group was in the alert range for insulin resistance [14,15]. No signs of muscle, pancreas, or kidney damage were observed. However, GGT level was elevated in the OB group, which indicates that these children had some liver injury or irritation.

Inflammation plays a crucial role in the development of obesity. A tendency toward decreased TNF-α levels and increased IL-10 levels in all groups after amaranth consumption was observed, which correlates with the reported anti-inflammatory effect of amaranth [7]. Increasing protein intake in meals helps lower body weight, improve cardiovascular health, and maintain normal kidney function [16]. Our data also showed that AmProt intake was safe, with the added benefit of lowering cholesterol and triglyceride levels and helping to reduce the inflammation state.

SCFA levels were elevated in children with obesity compared to those children with normal weight [17], which agrees with our results (Supplementary Figure S3). Higher levels of SCFAs may be implicated in gut dysbiosis and could be related to increased levels of leptin, but still the mechanism of action is not well understood [18]; however, increased levels of leptin are taken as a predictor of obesity in children [19].

The gut microbiota is a contributing factor in human obesity, but no studies have examined its modulation following amaranth protein consumption in humans. Obesity dysbiosis has been linked to an increase in Firmicutes levels, decrease in bacterial diversity, and changes in specific bacterial taxa [5,20]. No changes in richness and diversity in OW and OB were observed when compared to the NW group, but an increase was observed after AmProt consumption (Figure 2). A high abundance of Firmicutes in all groups was observed; however, there is no clear association between the Firmicutes/Bacteroidetes ratio and the obesity status [20,21]. Reports also indicate that a high abundance of Firmicutes and elevated TNF-α levels are associated with obesity [21,22], which aligns with our data. The high abundance of Firmicutes identified in this work may be related to individual dietary habits, as the gut microbiota is strongly associated with lifestyle, geography, and ethnicity [4]. A recent report demonstrated that the microbiota in obesity differs at country level; the microbiota is different between low-to-middle-income and high-income countries [23].

Increased abundance of *Collinsella* was observed only in the NW-AmProt group. *Collinsella* has been reported as a biomarker in Mexican adolescents with obesity, and it has also correlated with circulating insulin levels. Thus, this bacterium has emerged as a potential biomarker of probiotic efficacy [24,25]. *Bacteroides* was found in decreased abundance after AmProt in all groups. Some *Bacteroides* species are linked to lean and healthy phenotypes, whereas others are associated with increased risk of obesity [26]. *Anaerostipes* decreased in abundance in OW-AmProt and NW-AmProt but not in the OB groups, while *Coprococcus* increased its abundance in these groups (Figure 4). *Anaerostipes,* as an SCFA-producing bacterium, may influence gut health, but its role in obesity is poorly understood. The low abundance of the *Coprococcus* species, an SCFA-producing bacterium, has been linked to a higher incidence of obesity, T2DM, inflammatory bowel disease, and its reduction is correlated with depression [27]. In OB-AmProt, we observed an abundance increase in *Prevotella*, while *Faecalibacterium* was observed in OW-AmProt (Figure 4). Both *Prevotella* and *Faecalibacterium* have received significant attention for their potential roles in human health and for their strong associations with dietary patterns, including dietary fibers and plant-based foods [28,29]. The pro-inflammatory *Escherichia-Shigella* bacteria, which contribute to obesity development [30], were observed at higher abundance in the OW group but decreased after AmProt consumption.

OW was the group that showed the greatest changes in the gut microbiota, with very low abundances of *Blautia*, *Streptococcus*, *Fusicatenibacterium*, *Intestinimonas*, and *Eubacterium hallii*. *Blautia* is one of the most representative genera in the human intestinal microbiota (3 to 11%); however, its role in obesity remains ambiguous [31]. A decreased abundance of *Streptococcus* has been associated with obesity and T2D. *Fusicatenibacterium* is a bacterium associated with obesity, but conflicting results have been reported [17]. *E. hallii* is an important bacterium for the intestinal metabolic balance [32]. After AmProt consumption, the OW group showed an increase in *Butyricicoccus* and *Roseburia*. Some species of *Butyricicoccus*, a butyrate-producing bacterium, are being considered as next-generation probiotics [33], and some *Roseburia* species have shown health benefits by preventing intestinal inflammation [34]. *Ruminococcus* is a dominant gut bacterial in Mexican children with normal weight and shows low abundance in individuals with obesity [35], which agrees with our results (Figure 4). The abundance of the *Ruminococcus* genus was also positively correlated with protein intake [36]. We observed that some ASVs identified as *Ruminococcus* were highly increased in the NW-AmProt group.

One of the strongest positive correlations observed between metabolism and bacteria was the Pentose Phosphate Pathway (PPP) with *Blautia* (r = 0.852) and *Subdoligranulum* (r = 0.827). *Blautia* also showed a high correlation with glucuronate interconversion (r = 0.779). PPP and glucuronate interconversion were observed to be downregulated in mice fed an HFD [37,38]. PPP is the route for fermenting pentoses derived from fiber-rich diets and contributes to the production of SCFAs, which has been related to improved weight. However further studies should be directed toward analyzing in depth these metabolic changes and the gut microbiota.

## 5. Conclusions

This is the first report evaluating the effect of amaranth protein intake in children with different BMI values. A decrease in serum triglyceride and cholesterol levels was observed after AmProt consumption. A tendency toward a decrease in TNF-α and an increase in IL-10 levels in serum was observed. After AmProt consumption, changes in the gut microbiota were observed. Further work should be carried out to identify the gut microbiota at genus and strain levels to clarify which species/strains are beneficial or harmful in obesity development. In general, parents who supported their children in taking the AmProt daily reported an improvement in their children’s health. The work represents a prospective study of the effect of high-quality protein consumption on the gut microbiota in children. The strength of this study was that all the children were in the same municipality, with the same access to food and the same environmental conditions. The weakness lies in the small sample size, which limits the statistical robustness of the study. Further work should be carried out with a higher sample size, taking control over the diet and exercise that the children follow during the trial.

## Data Availability

The data generated and analyzed in this study are available upon request to the corresponding author.

## Supplementary Materials

The following supporting information can be downloaded at https://www.mdpi.com/article/10.3390/nu18111690/s1; Table S1: Proximal composition of amaranth flour and amaranth protein isolate. Table S2: Mineral composition in amaranth protein isolate Table S3: Serum biochemical profile in children with different body mass index. Table S4: Serum levels of interleukin and adipokine in children with different body mass index values. Table S5: MiSeq data sequencing. Table S6: The top 10 strongest bacteria-pathway correlations. Figure S1: Study design to analyze the effect of amaranth protein isolate consumption in the health status of children with overweight and obesity. Figure S2: (A) Electrophoretic pattern of amaranth protein isolate. (B) Dipeptidyl peptidase IV activity; (C) Angiotensin converting enzyme activity. Figure S3: Quantification of short-chain fatty acids in fecal matter before and after amaranth protein isolate consumption. Figure S4: Rarefraction curves. Figure S5: Spearman correlations between bacteria genus and metabolic pathways.
