# Supplementary material for: Dietary Supplementation with Amaranth Protein Isolate Modulates the Gut Microbiota in Children with Overweight and Obesity: A Nonrandomized Trial
Source: Nutrients. 2026 May 26;18(11):1690. doi: 10.3390/nu18111690 (PMC13258584; doi:10.3390/nu18111690)
Supplement: Supplementary file 1 [file nutrients-18-01690-s001.zip › nutrients-4234983-supplementary.pdf]

Supplementary information

Dietary supplementation with amaranth protein isolate modulates the gut microbiota in children with overweight and obesity: A nonrandomized trial

Supplementary Table S1. Proximal composition of amaranth flour and amaranth protein isolate

**Macromolecule**	**Flour**	**Isolated protein**
Protein[1]	15.3 ± 2.5	71.0 ±7.9
Fat	6.6 ±1.4	2.1 ±0.4
Ash	3.8 ±1.8	3.6 ±0.9
Fiber	3.4 ± 0.9	ND
Starch[2]	69.8 ±6.0	23.3 ±7.5

[1]Total Nitrogen x 6.25; [2] Determined as difference. ND=non determined

Supplementary Table S2. Mineral composition in amaranth protein isolate

**Metal**	Concentration (mg/kg)
Al	6.75
B	7.01
Ba	0.61
**Ca**	**450.9**
Cr	<0.05
Cu	11.1
Fe	13.1
K (%)	2.86
Li	<0.05
**Mg**	**455.1**
**Mn**	**8.7**
**Na**	**460.3**
P (%)	0.57
**Si**	**266.8**
Sr	2.32
**Zn**	**26.0**

Supplementary Table S3. Serum biochemical profile in children with different Body Mass Index

Parameter	Normal weight		Overweight		Obesity	
	**start n = 18**	**end n = 8**	**start n = 12**	**end n = 6**	**start n = 18**	**end n = 7**
**BMI** (kg/m^2)	16.7 ± 1.3*	17.3 ± 1.9	20.5 ±1.4*	22.1 ± 1.1	24.5 ± 2.7*	24.9 ± 2.9
**Glucose** (60-100 mg/dL)	88.5 ± 2.7	90.5 ± 5.6	92 ± 3.0	97 ± 2.6	96 ± 3.2	96 ± 4.0
**Cholesterol** (120-200 mg/dL)	184 ± 8.4	143 ± 13.7*	184 ± 10.6	147 ± 16*	196 ± 11.4	152 ± 14.1*
**Triglycerides** (<130 mg/dL)	113 ± 9.9	61 ± 13.9*	133 ± 18.7	100 ± 10.4	170 ± 15.1	122 ± 18.1*
**Uric acid** (2.5-5.5 mg/dL)	5.1 ± 0.4	4.2 ± 0.7	5.2 ± 0.3	3.9 ± 0.6	5.8 ± 0.4	5.6 ± 0.5
**Urea** (11-38.5 mg/dL)	19.9 ± 0.9	22.2 ± 3.4	22.8 ± 1.8	24.0 ± 2.5	21.4 ± 1.0	23.4 ± 1.8
**LDH** (100-630 U/L)	544 ± 21.4	442 ± 15.9	533 ± 23.9	432 ± 28.5	525 ± 25.1	489 ± 39.5
**Creatine kinase** (30-200 U/L)	168 ± 8.0	162 ± 30.1	155 ± 13.3	166 ± 6.8	141 ± 14.4	144 ± 29.1
**Amilase** (20- 100 U/L)	82 ±7.5	82 ± 13.7	72 ± 6.0	64 ± 6.3	82 ± 5.5	75 ± 8.3
**Lipase** (< 150 U/L)	53 ± 7.5	66 ± 9.8	52 ± 13.8	62 ±7.9	55 ± 8.3	50 ± 2.7
**Total protein** (6-7.8 g/dL)	8.1 ± 0.2	7.2 ± 0.2*	8.0 ± 0.2	7.3 ± 0.1*	8.2 ± 0.2	7.8 ± 9.3*
**Albumin** (3.8 – 5.4 g/dL)	5.5 ± 0.1	5.0 ± 0.2	5.5 ± 0.1	5.0 ± 0.1	5.5 ± 0.1	5.2 ± 0.2
**Alb/Glob ratio** 1.7-2.25	2.2 ± 0.1	2.4 ± 0.1	2.3 ± 0.1	2.3 ± 0.2	2.0 ± 0.05	2.1 ± 0.1
**AST** (10 – 50 U/L)	36.2 ± 2.1	31.3 ± 3.0	32.4 ± 1.8	32.3 ± 3.0	36.2 ± 3.8	38.0 ± 6.1
**ALT** (5- 45 U/L)	17.7 ± 1.6	17.9 ± 2.4	21.5 ± 3.7	19.3 ± 4.5	25.5 ± 3.6	31.4 ± 7.1
**ALP** (150 – 420 U/L)	773 ± 47.7	771 ± 86.1	730 ± 58.1	814 ± 86.3	697 ± 49.1	775 ± 82.3
**GGT** (< 20 U/L)	19.9 ± 1.9	22.4 ± 2.1	21.4 ± 1.1	17.8 ± 3.0	27.2 ± 2.9*	28.5 ±3.1*
**Total bilirubin** (< 1.0 mg/dL)	0.7 ± 0.04	0.6 ± 0.07	0.7 ± 0.05	0.6 ± 0.1	0.6 ± 0.05	0.5 ± 0.1
**Direct bilirubin** (<0.2 mg/dL)	0.2 ± 0.02	0.1 ± 0.04	0.2 ± 0.02	0.2 ± 0.05	0.2 ± 0.02	0.2 ± 0.03
**Indirect bilirubin** (<0.8 mg/dL)	0.5 ± 0.03	0.4 ± 0.04	0.5 ± 0.1	0.4 ± 0.1	0.4 ± 0.03	0.4 ± 0.1

Values in parentheses in the parameter's column correspond to normal values for children aged 8 to 12 years. LDH=lactate dehydrogenase; AST=aspartate aminotransferase; ALT=alanine amino transferase; ALP=alkaline phosphatase; GGT=gamma-glutamyl transferase. A one-way analysis of variance (ANOVA) and Tukey *post-hoc* analysis ($p<0.05$) were performed. The values are expressed as the mean ± standard error. Asterisk show a significant mean value among groups.

Supplementary Table S4. Serum levels of interleukin and adipokine in children with different Body Mass Index

Marker	Normal weight		Overweight		Obesity	
	start	end	start	end	start	end
**Insulin (µIU/mL)**	7.9 ± 1.9	8.0 ± 2.5	16 ± 9.7	14 ± 5.2	17.6 ± 8.8	20 ± 9.4
**Leptin (ng/mL)**	5.1 ± 2.7	5.0 ± 3.0	8.6 ± 3.0*	8.8 ± 1.8*	11.9 ± 2.3*	11 ±1.4*
**Adiponectin (µg/mL)**	4.8 ±2.3	5.1 ± 2.6	3.0 ± 1.0	2.8 ± 1.6	3.7± 2.5	4.9 ± 2.6
**TNFα (pg/mL)**	32 ± 19	28 ± 16	23 ± 11	18 ± 3.9	29 ± 15	24 ± 10
**MCP-1 (pg/mL)**	137 ± 89	149 ± 36	130 ± 74	211 ± 97	77 ± 45	202 ± 86*
**IL-1β (pg/mL)**	1.3 ± 0.7	1.4 ± 0.3	1.0 ± 0.4	1.4 ± 0.3	1.3 ± 0.1	1.4 ± 0.3
**IL-6 (pg/mL)**	0.8 ± 0.3	1.4 ± 1.2	1.1 ± 0.6	1.4 ± 0.5	1.3 ± 0.8	1.1 ± 1.0
**IL-10 (pg/mL)**	5.7 ± 3.0	8.5 ± 4.6	4.8 ± 2.3	9.1 ± 2.7	5.5 ± 4.2	7.0 ± 4.4

Values are expressed as the mean ± standard deviation. A Kruskal-Wallis test and Dunn's *post-hoc* analysis at $p<0.05$. Asterisk show a significant mean value among groups.

Supplementary Table S5. MiSeq data sequencing

Data	Normal weight		overweight		obesity	
	Start (n=7)	End (n=11)	Start (n=5)	End (n=5)	Start (n=12)	End (n=11)
Reads out	231,342	216,066	248,732	227,816	243,606	278,598
Denoised F	202,328	185,380	216,131	196,389	209,691	238,837
Denoised R	199,244	185,767	216,796	196,349	206,480	239,655
Merged	199,734	131,175	159,482	143,241	206,480	173,209
ASV	71,725	68,135	79,426	76,751	85,999	85,526

Supplementary Table S6. The top 10 strongest bacteria-pathway correlations

**Bacteria**	**Metabolic Pathway**	**Correlation Coefficient**
Blautia	Pentose phosphate pathway	0.852
Subdoligranulum	Pentose phosphate pathway	0.827
Blautia	Glucuronate interconversions	0.779
Bifidobacterium	Fructose and mannose metabolism	0.666
Anaerostipes	Butanoate metabolism	0.629
Subdoligranulum	ABC transporters	0.647
Dorea	ABC transporters	0.619
Agathobacter	Arginine biosynthesis	0.668
Erysipelotrichaceae UCG-003	Butanoate metabolism	0.586
Fusicatenibacter	Fructose and mannose metabolism	0.696

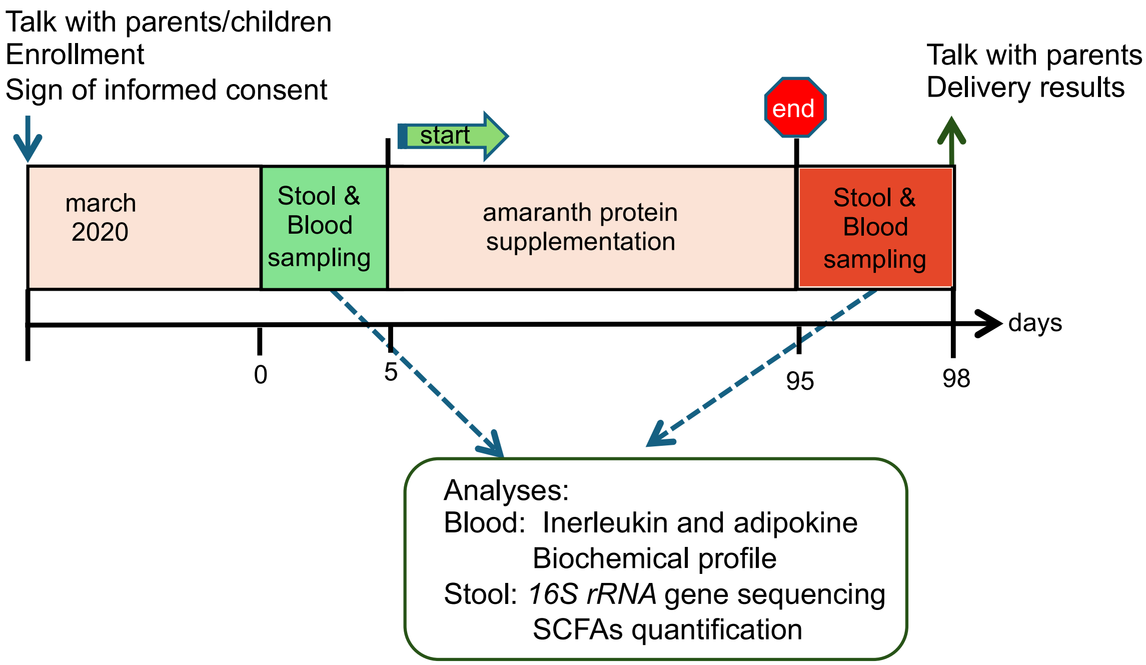

Supplementary Figure S1. Study design to analyze the effect of amaranth protein isolate consumption in the health status of children with overweight and obesity.

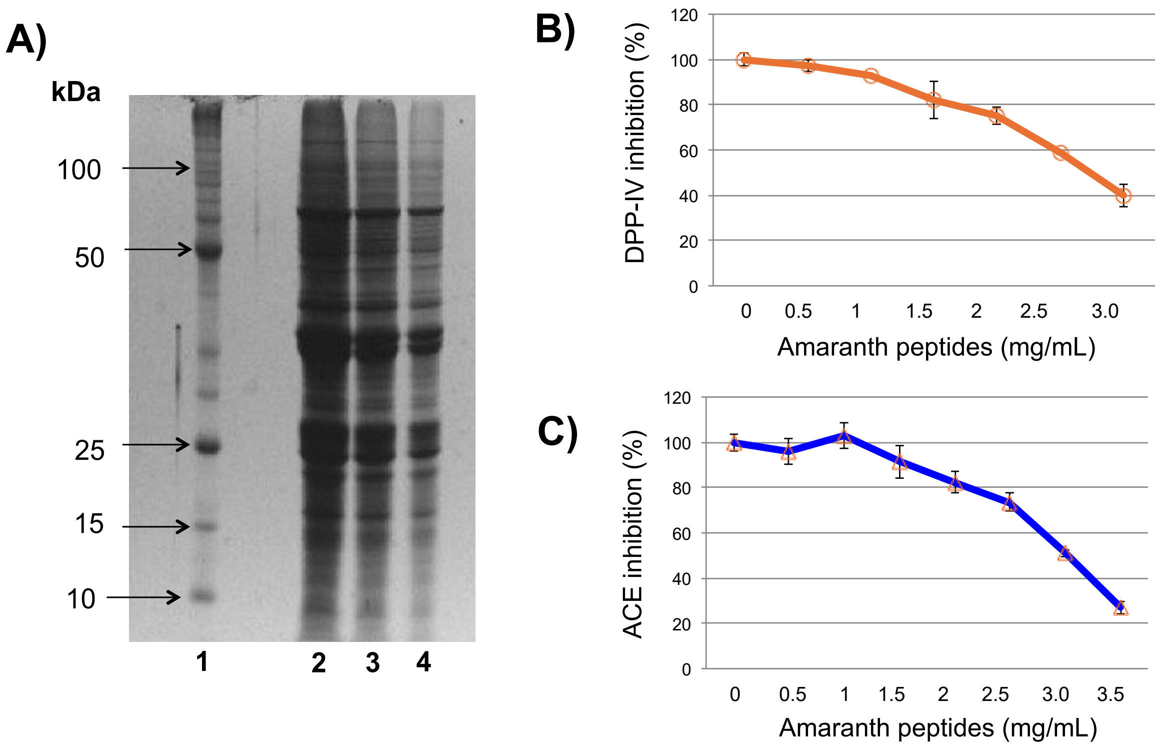

Supplementary Figure S2. A) Electrophoretic pattern of amaranth protein isolate (AmProt). Line 1=molecular weight marker; Lines 2-4=AmProt at different concentrations. Inhibitory activity of AmProt tryptic peptides against B) dipeptidyl peptidase-IV (DPP-IV) and C) angiotensin converting enzyme (ACE).

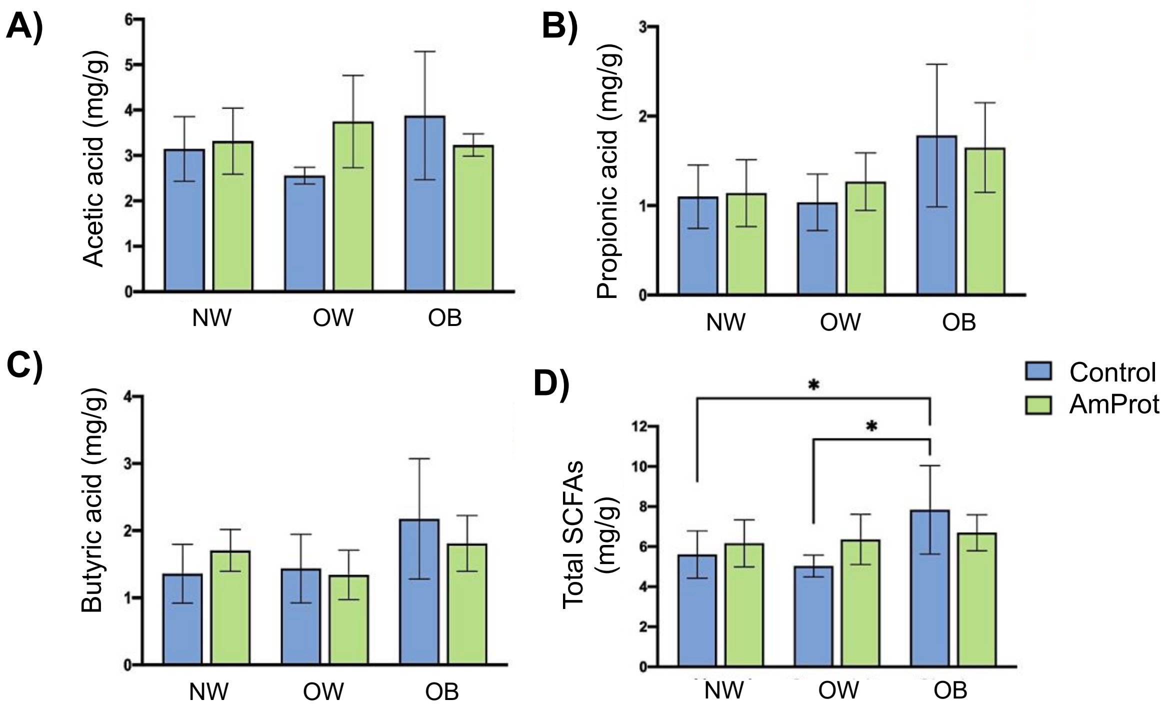

Supplementary Figure S3. Quantification of short-chain fatty acids (SCFAs) in fecal matter before and after amaranth protein-isolate (AmProt) consumption. A) acetic, B) propionic, C) butyric, and D) total SCFA. Columns express the mean values ± Standard Deviation. A Kruskal-Wallis test and Dunn's *post-hoc* analysis ($p<0.05$) were performed. Asterisk indicates the means with significant differences. Blue columns are groups at the beginning of the trial; green columns represent the same groups after 3 months AmProt consumption. NW=normal weight group; OW=overweight group; OB=obesity group.

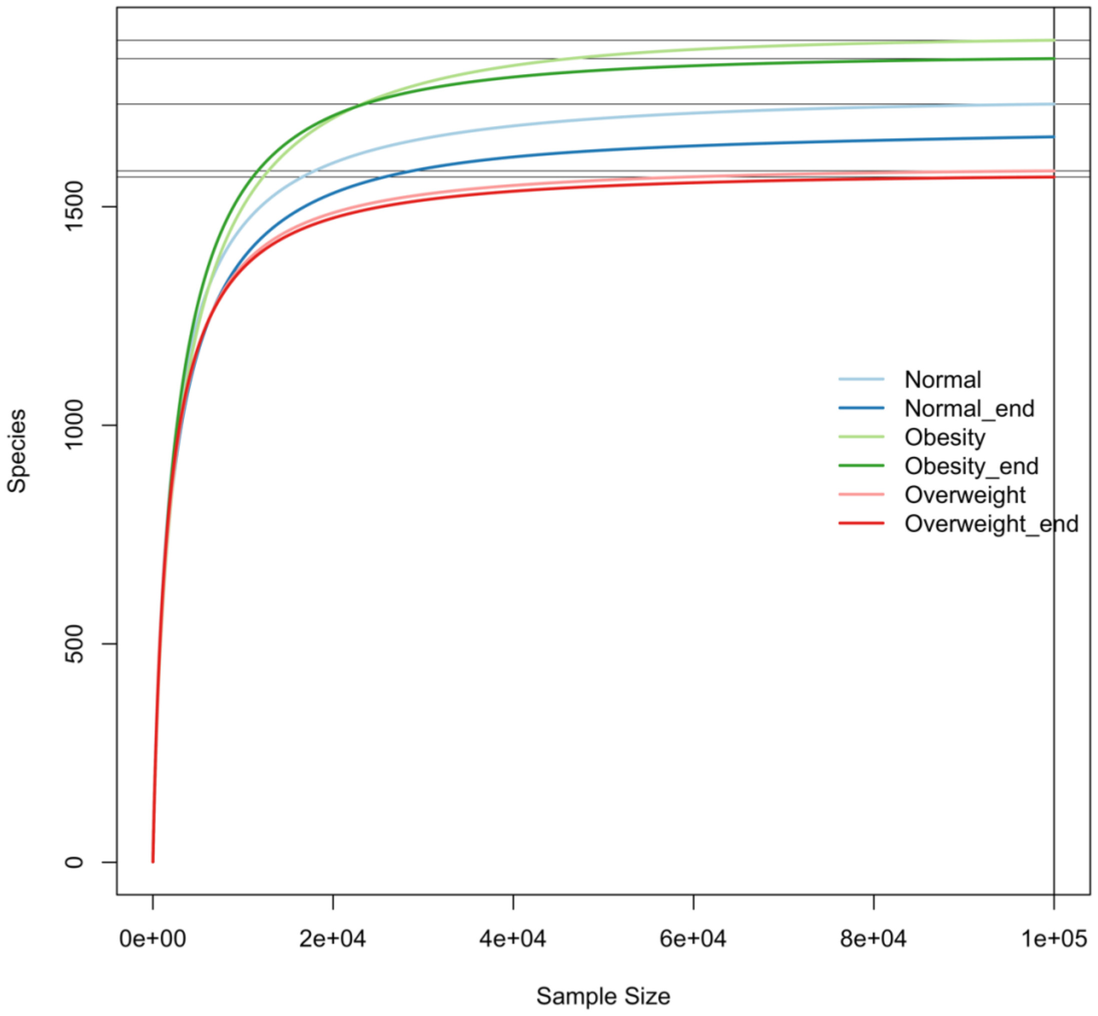

Supplementary Figure the S4. Rarefaction curves showing the observed species number in the normal weight, overweight and obesity groups before and after amaranth-protein isolate consumption. The y-axis shows the average numbers of ASVs per sample across group.

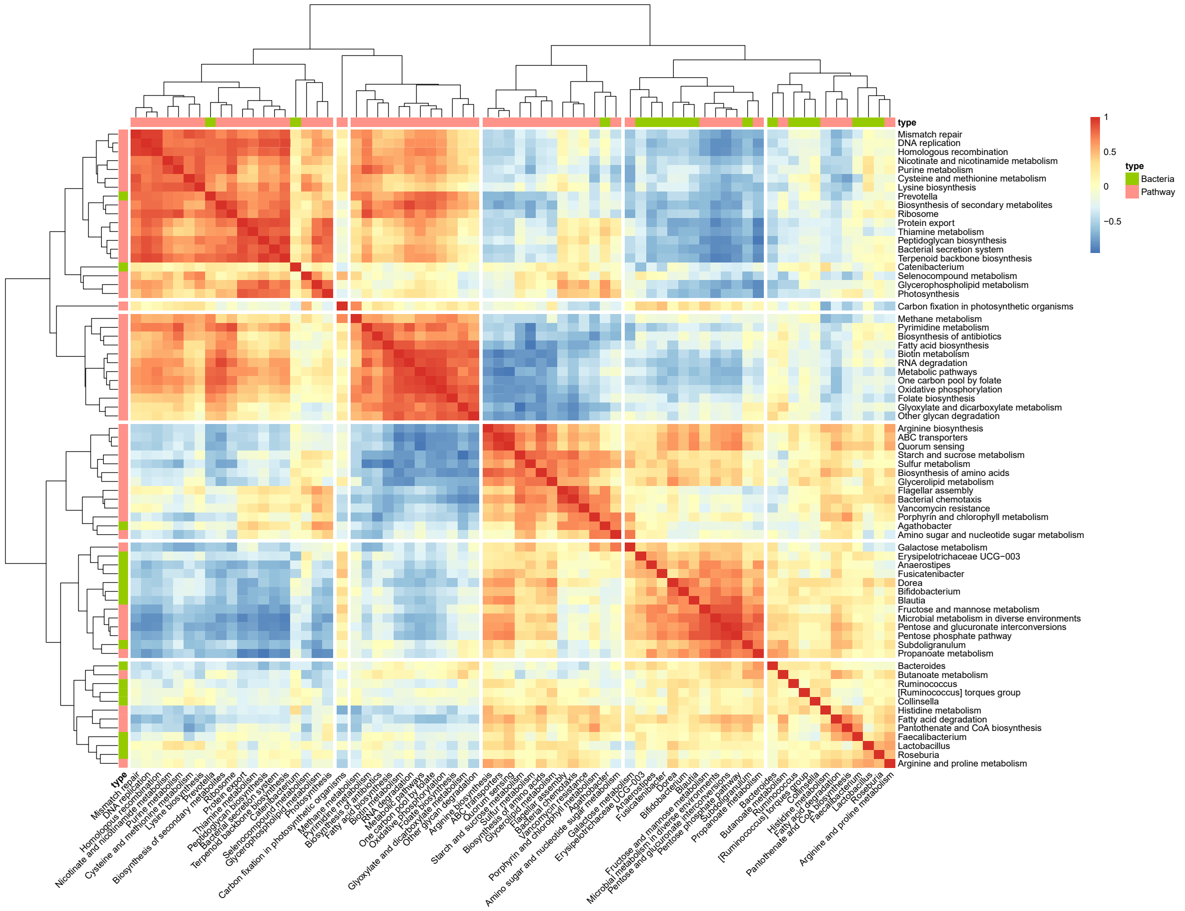

Supplementary Figure S5. Spearman correlations between bacteria genus and metabolic pathways. The type of variable is indicated in green for bacteria genera and pink for metabolic pathways.
